# Discrete-choice modelling of patient preferences for modes of drug administration

**DOI:** 10.1186/s13561-017-0162-6

**Published:** 2017-07-27

**Authors:** Ebenezer Kwabena Tetteh, Steve Morris, Nigel Titcheneker-Hooker

**Affiliations:** University College London, Gower Street, London, WC1E 6BT UK

**Keywords:** Discrete-choice experiments, Drug administration, Manufacturing, United Kingdom

## Abstract

The administration of (biologically-derived) drugs for various disease conditions involves consumption of resources that constitutes a direct monetary cost to healthcare payers and providers. An often ignored cost relates to a mismatch between patients’ preferences and the mode of drug administration. The “intangible” benefits of giving patients what they want in terms of the mode of drug delivery is seldom considered. This study aims to evaluate, in monetary terms, end-user preferences for the non-monetary attributes of different modes of drug administration using a discrete-choice experiment. It provides empirical support to the notion that there are significant benefits from developing patient-friendly approaches to drug delivery. The gross benefits per patient per unit administration is in the same order of magnitude as the savings in resource costs of administering drugs. The study argues that, as long as the underlying manufacturing science is capable, a patient-centred approach to producing drug delivery systems should be encouraged and pursued.

## Introduction

A recent systematic review [1] notes that administration of multiple drug doses over time requires different types of medical resources and hence can have a non-trivial impact on the monetary costs of healthcare delivery. This argument, however, does not consider the non-monetary hedonic characteristics (attributes) of administering drugs that are linked to the preferences of patients for different modes of drug administration. That is to say, a full accounting of the societal costs and benefits of resources expended on drug administration should take into account both the direct monetary and indirect non-monetary costs and benefits. This is because a given mode of drug administration that incurs the lowest monetary cost to healthcare payers or providers may incur hidden indirect costs in terms of a mismatch with what is preferred by end-users, be it patients or otherwise healthy people [2].

It is crucial therefore to understand and assess the characteristics (attributes) of drug administration – such that better drugs can be developed and manufactured tailored to patients’ preferences. The importance of this is evident from a report published by the Knowledge Transfer Network (HealthTech and Medicines) on “[t]he future of high value manufacturing [in the pharmaceutical, biopharmaceutical and medical device sectors] in the UK” [3]. The report identified, among a number of factors, the importance of: (1) early consideration of manufacturing needs, (2) flexible production facilities; (3) reducing cost to the UK National Health Services [NHS] and (4) the delivery of better services and improved health outcomes to patients. The last, in particular, focussed on the need for more stable, effective medicines; novel ways of administering them; and smart [packaging] technologies to monitor usage by patients. A full understanding of end-user preferences for modes of drug administration is necessary if objective (4) above is to be achieved.

In this study, we aim to evaluate the attributes of drug administration, focusing on the preferences of patients, or otherwise healthy people from the UK general public. We do this using a discrete choice experiments (DCE) that – in contrast to interviews, focus groups and other in-person surveys – supports quantitative estimation of the strength of end-user preferences for different attributes and how they are traded off against one another. In addition, a DCE supports monetary valuation of different attribute combinations that produce estimates of the indirect benefits to end-users. By monetizing preferences, a comparison with the direct monetary costs of administering biologic drugs can be made. The direct monetary costs of administration can be predicted using the algorithm developed by Tetteh and Morris [4]. And we will argue that these predicted cost estimates plus preference valuations measured in this paper should be considered in (bio)manufacturing decision-making. This is the kind of information manufacturers need in order to make patient-friendly drugs that have low administration costs. The use of such evidence in pre-market R&D and manufacturing decisions should yield drug products with added value.

Cost-effectiveness assessments, from a healthcare payer perspective, will capture the added value in terms of savings in drug administration costs. This is not the case for indirect end-user benefits. The comfort of an improved mode of drug administration is seldom considered in evidence-based medicine and often seen as “luxury”. The DCE conducted in this study however shows the monetary value of intangible end-user benefits (what some may consider luxury that can be ignored) is significant. This finding is most relevant to (bio)pharmaceutical manufacturers as they are the translators of promising (biologic) drug candidates into medicines that offer positive direct health benefits relative to placebo or existing treatments. The pertinent issue here is whether healthcare payers recognize and are willing to pay for the indirect benefits to end-users, besides the direct health benefits. If they do, manufacturers will be faced with the right incentives to produce drugs that make significant contribution to patient care.

## Background

Our starting point was the observation that drug administration is one part of the whole packaged good or service we call health or medical care. Preferences for different modes of drug administration reflect a derived demand for the direct health benefits offered by a given drug product. The mode of drug administration simply constitutes a vehicle via which these direct health benefits are delivered to a patient. The willingness to pay for a marketed drug product will include valuations of the direct health benefits it offers plus valuations of the means by which these direct health benefits are delivered. A DCE in which drug products are identical in every aspect except their mode of administration, provides the means of evaluating the attributes of drug administration separately. The attributes and attribute-levels chosen in such a DCE should be realistic and relevant to manufacturing decisions and/or consumer (end-user) choices.

Under the premise of utility-maximizing behaviour, an end-user (indexed *s*) will choose a given mode of administering a drug if the utility derived from that choice is the maximum among *J* alternative ways of administering that same drug. This represents *J* differentiated product versions of the same drug. Following Manski [5], the utility (${U}_{sj}^{\ast }$) from choosing alternative *j*(=1, 2, …, *J*) from among a set of *J* discrete products has: one, a systematic, explainable or observable component, *V*_*sj*_ that is a function of the attributes of drug administration; and two a random unexplainable error term, *ϵ*_*sj*_. We can write the following:

1 $$ \begin{array}{l}{\mathrm{U}}_{sj}^{\ast }={\mathrm{V}}_{sj}\left({\boldsymbol{\upbeta}}_{jk}{\mathbf{X}}_{jk}\right)+{\upepsilon}_{sj}\\ {}{\boldsymbol{\upbeta}}_{jk}{\mathbf{X}}_{jk}={\upbeta}_{\mathrm{k}}^{\hbox{'}}{\mathrm{X}}_{\mathrm{k}}^{\hbox{'}}+{\upbeta}_{\mathrm{p}}{\mathrm{C}}_{\mathrm{p}}+{\sum}_{\mathrm{j}=1}^{\mathrm{J}-1}{ASC}_{\mathrm{j}}\end{array} $$

where ***X***_*jk*_ is a vector of attribute-levels decomposed into ${X}_k^{\prime }$, a vector of generic non-monetary attribute-levels and *C*_*p*_, the cost associated with alternative *j*. ***β***_*jk*_is a vector of preference coefficients, decomposed into ${\beta}_k^{\prime }$, a vector of coefficients for the non-monetary attributes and *β*_*p*_, coefficient for the cost attribute. The random error term (*ϵ*_*sj*_) could refer to effects of unobserved attributes; imperfect information on alternative products available; measurement error; misspecification of the utility function; heterogeneity in preferences or simply random behaviour [6]. *ASC*_*j*_ is an alternative-specific-constant to capture peculiar effects of each alternative product that are not reflected in the attributes. ($\sum_{\mathrm{j}=1}^{\mathrm{J}-1}{ASC}_{\mathrm{j}}$ may be considered as the mean of *ϵ*_*sj*_.)

Given a sample of end-users (*S*), a number of choice sets or situations (*N*) faced by each end-user and prior knowledge of the ***β***_*jk*_vector, the probability (*P*) that product *j*(=1) will be chosen above the other *J* – 1 products can be estimated using the basic multinomial logit (MNL) model [7] as:

2 $$ \begin{array}{l}{\mathrm{P}}_{1\mathrm{ns}}\left({\mathrm{y}}_1=1\right)={\mathrm{P}}_{1\mathrm{ns}}\left({U}_1^{\ast }>{U}_j^{\ast}\right)=\frac{ \exp \left(\upmu \left[{\mathrm{V}}_{1\mathrm{ns}}\right]\right)}{\sum_{\mathrm{j}=1}^{\mathrm{J}} \exp \left(\upmu \left[{\mathrm{V}}_{jns}\right]\right)}\\ {}\mathrm{D}\left({\upepsilon}_{sj}\right)= \exp \left(-{\upepsilon}_{sj}\right) \exp \left(- \exp \left(\upmu {\upepsilon}_{sj}\right)\right);\mathrm{F}\left({\upepsilon}_{sj}\right)= \exp \left(- \exp \left(\upmu {\upepsilon}_{sj}\right)\right)\end{array} $$

where *y* denotes the choices made such that *y*_1_ = 1 if product *j*(=1) is selected and zero otherwise. The term *μ* is a positive scale parameter that is inversely related to the error variance (${\sigma}_{\epsilon}^2$) of the panel of choices made.

Equation (2) requires *D(ϵ*_*sj*_ *)*, a Gumbel (log-Weibull) probability density function for independent and identically distributed (IID) error terms, where *F(ϵ*_*sj*_ *)* is the corresponding cumulative density function. Since the error terms are specific to each choice dataset, *μ* is usually normalized to one for the basic MNL model – indicating homoskedastic (constant) error variance. With preference coefficients fixed or invariant over end-users, and IID error terms, we have the so-called independence from [ir]relevant alternatives (IIA) assumption – which suggests the ratio of choice probabilities is independent of the inclusion or omission of other products.

## Methods

### Attributes, attribute-levels and experimental designs

In conducting our DCE, we first set out to identify a common set of relevant attributes and attribute-levels for different modes of drug administration. We do this via a selective review of literature investigating different modes of administering drugs [8–14]. Table 1 below shows our selected set of attributes, definitions of these attributes and their levels.

**Table 1:** Attributes, definitions and attribute-levels

Attributes	Definitions	Levels
Method of drug administration	This attribute refers to the route by which therapeutically-active drug products are physically administered into a patient. The attribute-levels include all other “needle-free” methods of drug administration to capture the preferences of patients who desire oral drug delivery and/or have a fear of needles.	1. Intravenous delivery 2. Subcutaneous delivery 3. Intramuscular delivery 4. Needle-free delivery
Dosing frequency	This attribute refers to the frequency of administering a drug for a single full course of treatment. Dosing frequency associated with repeated treatments should not be considered.	1. Once every six months 2. Once every month 3. Once every week 4. Once every day
Setting	This attribute refers to place (clinical and non-clinical settings) where a given drug is administered. Clinical settings include, for example, hospitals, outpatient clinics, care homes, offices of general practitioners/physicians etc. Non-clinical settings include home, schools and other public places.	1. Clinical 2. Non-clinical + self-administration* 3. Non-clinical + supervision*
Disruption to daily activities	This attribute refers to how a given method of drug administration or dosing frequency disrupts the daily activities of patients. Disruptions could be due to, for example, repeated venepuncture and, in the extreme, immobility (hospitalization for the sole purpose of drug administration).	1. None 2. Moderate but manageable 3. Moderate but I can’t cope 4. Severe
Risk of adverse events	This attribute refers to features of drug administration that might cause discomfort or injury to patients or health-staff administering drugs. This could be local or generalized adverse events such as indurations; damage to nerves and blood vessels; abscess formation around the sites of injection etc. This is separate from side-effects of the drug molecule itself.	1. None 2. Moderate 3. Severe
Cost	This attribute refers to the additional time and travel costs borne out-of-pocket by the patient each time they have to take or their medicines or it has to be given to them by health workers.	1. £0 2. £10 3. £50 4. £100

*Notes*: * This refers to the situation where people, if properly trained, could self-administer the drug in a non-clinical setting; or otherwise, their medications will have to be delivered to them under the supervision of qualified healthcare professional, for example, a community or district nurse. Given this set of attributes and attribute-levels, we have a full factorial of 2304 (= 4^4^32) possible profiles or treatment combinations

Considering the mode of administration is simply a vehicle for delivering the direct health benefits offered by a drug to patients, we opted to specify the alternative products as drugs that are identical in every aspect apart from the manner in which they are administered to patients. We used a forced-choice format of presenting survey respondents with two unlabelled drugs *A* and *B*. We did not include an “opt-out” alternative as we found it difficult to imagine that people will choose not to have a clinically-beneficial drug simply because the way in which the drug is administered is not what they prefer.

The next step was to develop the experimental designs that will form the basis of our survey questionnaires. The designs were created from fractional-factorial designs for estimating only *main effects* of the attribute-levels. This was done in SAS v. 9.3, using a set of macros and programming codes written by Kuhfeld [15, 16], as follows. We first used the macro “MktRuns” to gain some insights as to the appropriate number of runs, i.e., the sizes of candidate set-designs we could use. The “MktRuns” macro suggested (among others) the following sizes: 48 runs (=24 choice sets), 72 runs (=36 choice sets) and 144 runs (=72 choice sets). We then used the macro “MkTex” to create corresponding candidate set-designs in 48, 72 and 144 runs. Using the macro “ChoicEff”, we identified and evaluated the statistical efficiency of the best experimental design containing 24 choice sets and drawn from these candidate set-designs. We found a 24 choice-set design with a relative D-efficiency of 65%. This was developed from the candidate set-design with 48 runs.

To test the integrity of the experimental designs above (since we had no prior information on the attribute coefficients), we merged them with simulated pre-pilot discrete-choice data using the macro “MktMerge”. Given the artificial dataset created, we estimated a basic MNL model using the macro “phChoice” and SAS PHREG procedure. Compared with the other competing 24 choice-set designs developed from candidate set-designs with 72 and 144 runs, the design developed from the candidate with 48 runs, produced the lowest estimates of standard errors over *all* attribute-coefficients for the same simulated choice data. It also had the highest number of statistically significant attribute-coefficients.^1^ We therefore chose this design for our survey questionnaire.

However, there is a trade-off here: an experimental design with the highest possible D-efficiency may impose greater cognitive burden (task complexity) on survey respondents. One has to balance a desire for near-optimal designs with the possibility of collecting irrational or inconsistent choices [17]. We therefore, using the macro “MktBlock”, partitioned our chosen experimental design into two versions – such that each block version contained a sequence of 12 randomly allocated choice tasks.

### Survey administration

**Fig. 1:**
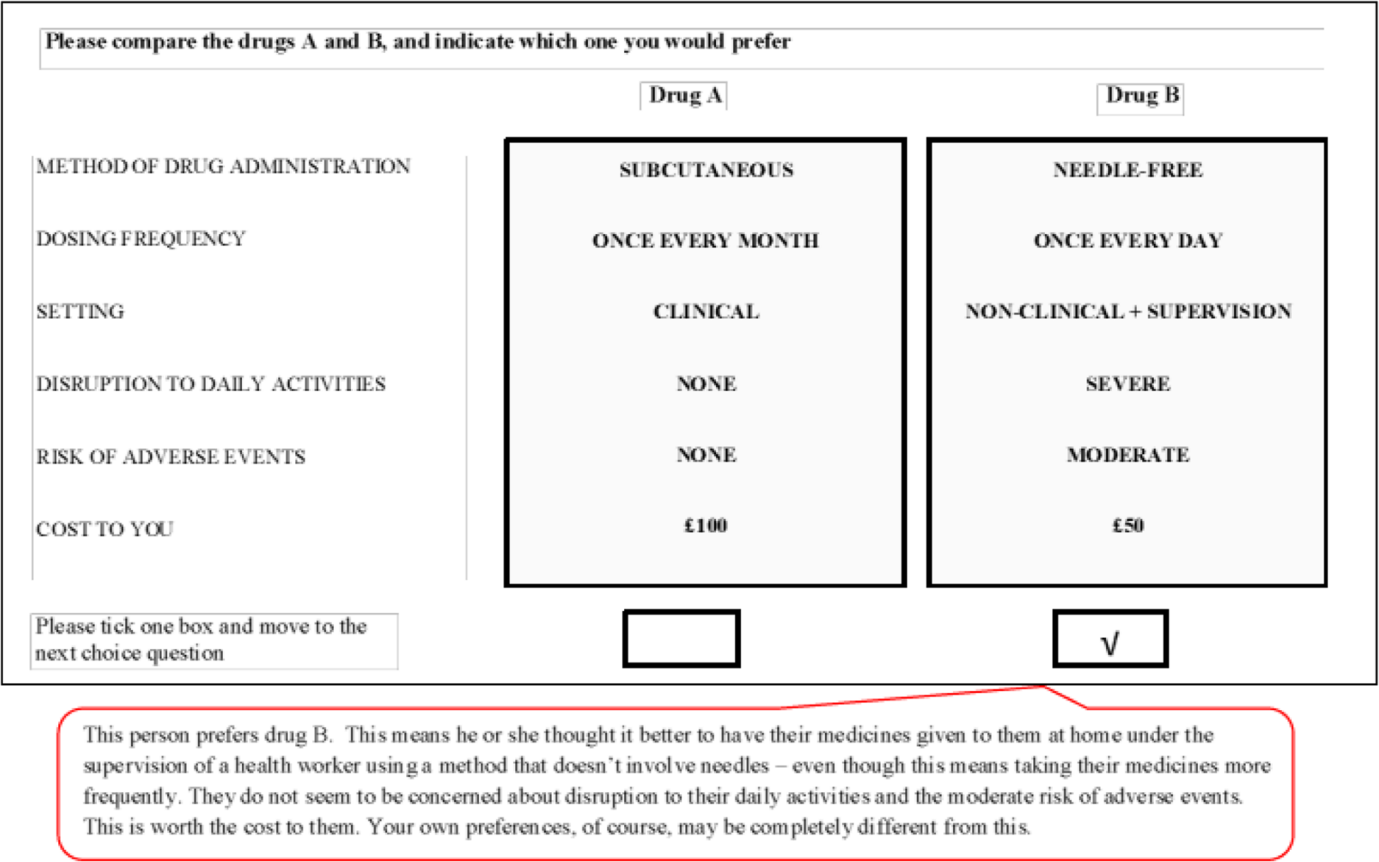
Example choice set

From the blocked experimental designs above, we developed two draft versions of the survey questionnaire. Each questionnaire was split into three sections. The first section provided a preamble with information about the purpose of the study and the hypothetical constructed context in which respondents had to make their choices. It also provided descriptions of the attributes and attribute-levels as well as an example of a completed choice set as a guide for the survey respondents (see Fig. 1 below). The second section contained the actual sequence of 12 choice questions or situations; and the third section collected anonymized information on individual respondents’ characteristics. We did not collect any data on respondents’ stated non-attendance to the attributes as this was not an objective of this study. The anonymized format of the questionnaires meant we did not require ethical approval prior to administering the survey.

Before sending the questionnaires out, we carried out a small-scale informal pilot of the draft versions of the questionnaires with no more than five people from the UK general public (given the time and resources available for this study). We asked recipients of the questionnaires to check the wording of the questionnaire; to ensure that the instructions were clear and to identify what might be perceived as implausible combination of attributes and attribute-levels. The respondents found all combinations of attributes and attribute-levels plausible, although some combinations may not be technologically feasible (given the current state of manufacturing or formulation science). We found that it took, on average, 15–20 min to complete each block version of the questionnaire. Following the pilot phase, we made small wording changes to the questionnaire to improve clarity.

We determined that a minimum sample size of 200 survey respondents will be adequate for meaning analyses. This was not derived from statistical sampling theory requiring accurate prior estimates of preference coefficients or choice probabilities/proportions. It is a pragmatic choice determined by the research budget and it is consistent with the range of sample sizes reported in Bridges et al. [17]. With the help of a commercial vendor (Survey Monkey), the questionnaires were administered online to a sample of people from the UK general population. It took roughly 2 weeks for the vendor to complete the web-based surveys.

### Econometric modelling

To explore plausible explanations (unobserved heterogeneity, variation in preferences, respondent fatigue etc.) for the observed sequence of choices in the data collected, we estimated a number of econometric models. As our starting point, we estimated a basic MNL model. The IID/IIA assumption underlying this model (with normalization of the scale parameter to one), however, is equivalent to saying that all survey respondents have the same preferences and/or that unobserved variation around these preferences are similar. For this reason, some researchers will argue that all estimates derived from the basic MNL model are biased. We therefore considered alternative econometric models that relax the IID/IIA restriction.

We considered a heteroskedastic multinomial (HMNL) model, where the scale parameter is no longer normalized to one but considered a variable that must be estimated. The error terms are therefore no longer IID distributed, and a typical approach is to express the scale parameter as a function of a vector of respondents’ characteristics (**Z**). The probability of an individual choosing alternative product *j* from among a set of competing products, in a given choice situation, is then given by:

3 $$ {\mathrm{P}}_{jns}\left({\mathrm{y}}_{\mathrm{j}}=1\right)=\frac{ \exp \left( \exp \left(\upalpha {\mathbf{Z}}_{\mathrm{s}}\right){\upbeta}_{jk}{\mathbf{X}}_{jk}\right)}{\sum_{\mathrm{j}=1}^{\mathrm{J}} \exp \left( \exp \left(\upalpha {\mathbf{Z}}_{\mathrm{s}}\right){\upbeta}_{jk}{\mathbf{X}}_{jk}\right)} $$

where *α* is a vector of coefficients reflecting the influence of respondents’ characteristics on the error variance. If $\widehat{\alpha}$ is not statistically different from zero, we revert back to or close to the basic MNL model. If $\widehat{\alpha}$ is statistically significant different from zero, then it is possible to *exogenously* determine subpopulations with somewhat identical preferences [18].

A variant of the HMNL model is the entropy multinomial (EMNL) model in which the scale parameter is a function of entropy (*E*): a measure of the information content or uncertainty represented in the probability distribution of a discrete random variable, in this case the choice variable *y*. In DCE literature, entropy summarizes the impact of task complexity or respondent fatigue due to the number of choice alternatives; the number and correlation between attributes and attribute-levels; and similarity between the alternatives. The relationship between the scale parameter and entropy of each choice situation can be expressed as:

4 $$ \begin{array}{l}{\upmu}_{\mathrm{ns}}= \exp \left({\uptheta}_1{\mathrm{E}}_{\mathrm{ns}}+{\uptheta}_2{\mathrm{E}}_{\mathrm{ns}}^2\right)\\ {}{\mathrm{E}}_{\mathrm{ns}}=-{\sum}_{\mathrm{j}=1}^{\mathrm{J}}\widehat{{\mathrm{P}}_{jns}} \log \left(\widehat{{\mathrm{P}}_{jns}}\right)\end{array} $$

where $\widehat{P_{jns}}$ is the estimated choice probability from the basic MNL model; *θ*_1_ , *θ*_2_ are parameters associated with entropy. The linear term $\widehat{\theta_1}$ measures deviation from maximum entropy, i.e., completely random choices whilst the quadratic term $\widehat{\theta_2}$ identifies non-linearity in the relationship above. The case of $\widehat{\theta_1}>0$ indicates entropy is either offset by exertion of more effort and/or (independent of effort) respondents’ under-estimation of the differences between choice alternatives. Researchers often treat the case of $\widehat{\theta_1}<0$ and $\widehat{\theta_2}>0$ as indicative of respondent fatigue (declining effort) as a survey respondent works through a sequence of choice sets [19, 20].

Next we considered the mixed multinomial (MMNL) model in which ***β*** varies randomly across individual respondents. Typically, these random coefficients are drawn from a mixture of continuous parametric distributions denoted by *f*(*β*∣*δ*), where *δ* refers to parameters of that mixture distribution. The choice probability for alternative product *j* (out of all *J* products) is then given by:

5 $$ {\mathrm{P}}_{jns}\left({\mathrm{y}}_{\mathrm{j}}=1\right)={\int}_{\upbeta_{sjk}}\left[\frac{ \exp \left({\upbeta}_{sjk}{\mathbf{X}}_{jk}\right)}{\sum_{\mathrm{j}=1}^{\mathrm{J}} \exp \left({\upbeta}_{sjk}{\mathbf{X}}_{jk}\right)}\right].\mathrm{f}\left(\upbeta |\updelta \right)\partial {\upbeta}_{sjk} $$

where the values of *β*_*sjk*_ are drawn from the continuous mixture distribution *f*(*β*∣*δ*) [21]. Here we assumed the individual-specific non-price preference coefficients that had no statistically significant effects in the basic MNL model are normally distributed and correlated with a price coefficient that is log-normal distributed and constrained to be negative. This combination of random attribute-coefficients and extreme-value (Gumbel) distributed error terms, however, means the MMNL model cannot be solved analytically but approximated via simulations with a finite number of draws.

Finally, we considered a latent-class multinomial (LCMNL) model that assumes attribute-coefficients are drawn from a mixture of non-parametric discrete distributions, representing *C* latent classes of homogenous subpopulations. It is not known a priori which latent class an individual belongs to; and the probability of latent-class membership (*π*) can be estimated as:

6 $$ {\uppi}_{cs}\left(\upgamma \right)=\frac{ \exp \left({\nu}_c+{\upgamma}_{\mathrm{c}}{\mathbf{Z}}_{\mathrm{s}}\right)}{1+{\sum}_{\mathrm{c}=1}^{\mathrm{C}-1} \exp \left({\nu}_c+{\upgamma}_{\mathrm{c}}{\mathbf{Z}}_{\mathrm{s}}\right)} $$

where $\sum_{c=1}^C{\pi}_c=1$, the vector *γ* (=*γ*_1_, *γ*_2_,  … , *γ*_*C*_) refers to the effect of individuals’ characteristics on class membership, and *ν*_*c*_ is a vector of class-specific constants [22]. Unconditional on class membership, the probability (*P* ^*^) of observing the sequence of *N* choices that an individual respondent makes is given by:

7 $$ {\mathrm{P}}^{\ast}\left({\mathrm{y}}_{Ns}=1\right)=\sum_{\mathrm{c}=1}^{{\mathrm{C}}^{\ast }}{\uppi}_{cs}\prod_{\mathrm{n}=1}^{\mathrm{N}}\prod_{\mathrm{j}=1}^{\mathrm{J}}{\left(\frac{ \exp \left({\upbeta}_{cjk}{\mathbf{X}}_{jk}\right)}{\sum_{\mathrm{j}=1}^{\mathrm{J}} \exp \left({\upbeta}_{cjk}{\mathbf{X}}_{jk}\right)}\right)}^{{\mathrm{y}}_{\mathrm{s}}} $$

where the optimal number of latent classes *C* ^*^ is determined by: (1) estimating a series of LCMNL models with different numbers of latent classes; (2) choosing the preferred model using the lowest consistent Akaike Information Criterion [cAIC] and/or Bayes Information Criterion [BIC]; and (3) making judgements on the trade-off between improved log-likelihoods and increase in standard errors (loss in precision) of the attribute-coefficients as the number of latent-classes gets large.

We estimated the models in STATA v. 11 using the attribute-based (**X**) and respondent-characteristics (**Z**) variables in Table 2 below. The **Z** variables are those in the shaded region of Table 2. The values of our explanatory variables are effects coded rather than 0–1 dummies to ensure the alternative-specific constant and other constant terms carry no information about the reference or omitted categories. See Bech and Gyrd-Hansen [23].

**Table 2:** Explanatory variables

Variables	Definitions (Effects coding)
INTRAVENOUS	=1 if a drug is administered intravenously (1, 0, 0, −1)
SUBCUTANEOUS	=1 if a drug is administered subcutaneously (0, 1, 0, −1)
INTRAMUSCULAR	=1 if a drug is administered ntramuscularly (0, 0, 1, −1). The reference category (−1) is administration via needle-free routes
DOSFREQ	Continuous variable referring to the number of unit administrations over a one year period for a single full course of treatment
NONCLINICAL_SELF	=1 if a drug is self-administered in non-clinical settings (1, 0, −1)
NONCLINICAL_SUPV	=1 if a drug is administered in non-clinical settings under the supervision of a qualified healthcare professional (0, 1, −1). The reference category (−1) is drug administration in clinical settings
DDA_MODERATE1	=1 if a given mode of administration is associated with moderate but manageable disruption to respondents’ daily activities (1, 0, 0, −1)
DDA_MODERATE2	=1 if a given mode of drug administration is associated with moderate disruptions to daily activities that a respondent cannot cope with (0, 1, 0, −1)
DDA_SEVERE	=1 if a given mode of drug administration is associated with severe disruption to the respondent’s daily activities (0, 0, 1, −1). The reference category (−1) is a mode of administration that carries no risk of disruption to patients’ daily activities
RAE_MODERATE	=1 if the risk of adverse events associated with a given mode of drug administration s moderate (1, 0, −1)
RAE_SEVERE	=1 if the risk of adverse events associated with a given mode of drug administration is severe (0, 1, −1). The reference category (−1) is drug delivery that is associated with no risk of adverse events
COST	Continuous variable indicating the time and travel costs borne by patients per unit administration
A	Alternative-specific constant = 1 for drug option *A* (1, −1). The reference point, drug option *B* = −1
FEMALE	=1 if survey respondent is female (1, −1). The reference category (−1) are males
RESPONDENTAGE	Continuous variable indicating the age of a survey respondent
VOCATIONAL	=1 if the highest level of education attained by a respondent is vocational training (1, 0, −1)
GCSEs_O + A	=1 if the highest level of education attained by a respondent is GCSEs O′ and A’ levels (0, 1, −1). The reference category (−1) are respondents with “higher education”
EMPLOYED	=1 if respondent is employed (1, −1). The reference category (−1) are those who are currently unemployed
INCOME_1	=1 if respondent’s annual household income is under £15,000 (1, 0, 0, 0, −1)
INCOME_2	=1 if respondent’s annual household income is between £15,000 and £29,999 (0, 1, 0, 0, −1)
INCOME_3	=1 if respondent’s annual household income is between £30,000 and £49,999 (0, 0, 1, 0, −1)
INCOME_4	=1 if respondent’s annual household income is between £50,000 and £75,000 (0, 0, 0, 1, −1). The reference category (−1) are respondents with annual household income in excess of £75,000
PRIOR_ ILLNESS	=1 if a survey respondent had received medical treatment under the advice or guidance of a qualified health worker over the past year (1, −1). The reference category (−1) are those who remained healthy over the past year

### Measuring patient benefits

The outputs of the models above allow us to compute, first, the marginal willingness-to-pay (MWTP) for a given change in an attribute (level) or a bundle of attributes. MWTP is the marginal rate of substitution between the non-monetary attributes (singly or in a bundle) and the price/cost attribute – assuming there is only one product available that will be chosen with a 100% certainty. For our purposes, we only computed $\widehat{\mathrm{MWTP}}$ for a *single* non-monetary attribute (= $-\widehat{\upbeta_{\mathrm{k}}}/\widehat{\upbeta_{\mathrm{p}}}$). Classical confidence intervals were generated using 100 bootstrap replicates of $\widehat{\mathrm{MWTP}}$. Admittedly, a higher number of replicates is needed for more precise estimation but we prefer this procedure as it is: (1) computationally less demanding; (2) uses actual data from respondents without making parametric assumptions about the distribution of $\widehat{\mathrm{MWTP}}$ [24]; and (3) compatible with all STATA estimators for the HMNL, EMNL, MMNL and LCMNL models.

Second, we estimated the incremental welfare gain or loss from switching (changing) from one product to another using the expected compensating variation (ECV). This is a more valid measure of welfare benefits when there is uncertainty about which product will be chosen. For discrete-choice probabilities estimated using an MNL-type model, ECV is formally computed as follows:

8 $$ \widehat{ECV}=-\frac{1}{\widehat{\uplambda}}\left[ \ln \sum_{\mathrm{j}=1}^{\mathrm{J}} \exp \left(\widehat{{\mathrm{V}}_{\mathrm{j}}^0}\right)- \ln \sum_{\mathrm{j}=1}^{\mathrm{J}} \exp \left(\widehat{{\mathrm{V}}_{\mathrm{j}}^1}\right)\right] $$

where λ is the marginal utility of income proxied by the negative coefficient of the price/cost attribute; and the superscripts 0 and 1 denote the conditions before and after the change (switch). The log-sum expressions or “inclusive values” in the brackets effectively weight the systematic utilities by the probability that an alternative product will be chosen in each state. Analogous to a change in consumer surplus, ECV measures the amount of money that will have to be extracted from an individual for them to remain indifferent between the initial (0) and final (1) states [25–27].

The ECV estimates computed in this study thus provides a monetary value of what might be considered intangible benefits of giving patients what they want in terms of the mode of drug delivery. From previous work [4], however, we know there are potential administration-cost savings to healthcare payers and providers from reformulating or reverse-engineering a drug product. To ascertain whether these intangible benefits are of any significant importance, we compared our ECV estimates with predicted administration cost savings of switching from one mode of drug delivery to another.

## Results

### Descriptive statistics

On completion of the DCE survey, we checked the data collected for incomplete sequence(s) of choices so as to avoid estimation biases due to discontinuous preferences (“noise”) created by information overload, boredom, unfamiliarity with or lack of interest in the survey. We found that each survey respondent completed all 12 choice tasks. Our estimation sample thus provided 10,608 usable choice responses from 442 respondents. We had no information on the number of people the vendor approached in order to achieve the minimum number of respondents. It is not possible therefore to compute response rates for the survey although the choice data was collected from more than the minimum number of respondents specified. Table 3 provides a summary of the sample demographic characteristics. We make no argument that this sample is representative of the UK population.

**Table 3:** Demographic characteristics of sample

Characteristics (of 442 respondents)	N (% of sample)
Gender	
Male*	181 (40.95)
Female	261 (59.05)
Respondents’ age	
17–34 years	151 (34.16)
35–49 years	155 (35.07)
≥ 50 years	136 (30.77)
Employment status	
Employed	307 (69.46)
Unemployed*	135 (30.54)
Household-income category	
< £15,000 (per year)	109 (24.66)
£15,000 – £29,999 (per year)	134 (30.32)
£30,000 – £49,999 (per year)	118 (26.70)
£50,000 – £75,000 (per year)	52 (11.76)
> £75,000 (per year)*	29 (6.56)
Highest education achieved	
GCSEs O & A levels	189 (42.76)
Higher education*	212 (47.96)
Vocational training	41 (9.28)
Prior illness (in the past year)	
Yes	178 (40.27)
No*	264 (59.73)

*Notes*: N = number of respondents; * indicates the reference category for the effects coding used (see also Table 2)

### Factors influencing choices

Table 4 shows the results from our econometric modelling. Given that we employed a forced-choice format for our survey with only two alternatives, we could not perform a Hausman-McFadden [28] statistical test for the IID/IIA assumption underlying the basic MNL model. However, the different results obtained from HMNL, EMNL, MMNL and LCMNL models suggest that the IID/IIA assumption would have been violated. The HMNL and EMNL models indicate non-constant error terms, whilst the MMNL and LCMNL models indicate variation in observed preferences across the survey respondents. Based on the log-likelihoods and Akaike Information Criterion (AIC), the LCMNL model with two latent classes^2^ offers the best model fit to our data. That is to say, preference variation in our dataset can be conveniently represented by two homogenous subgroups of respondents.

**Table 4:** Econometric results

Dependent variable: CHOICE PROBABILITY						
	MNL model	HMNL model	EMNL model	MMNL model	LCMNL model	
Variables/Coefficients:	$\widehat{\upbeta}(SE)$	$\widehat{\upbeta}$(SE)	$\widehat{\upbeta}(SE)$	$\widehat{\upbeta_{\mathrm{s}}}(SE)$	${\widehat{\upbeta}}_1(SE)$	${\widehat{\upbeta}}_2(SE)$
INTRAVENOUS	−0.011 (0.091)	0.005 (0.057)	0.017 (0.042)	−0.026 (0.110)	−0.119 (0.439)	0.021 (0.123)
SUBCUTANEOUS	0.021 (0.043)	0.016 (0.027)	0.002 (0.019)	−0.016 (0.049)	−0.073 (0.130)	0.070 (0.060)
INTRAMUSCULAR	−0.136 (0.049)^**^	−0.090 (0.031)^**^	−0.111 (0.037)^**^	−0.123 (0.053)^*^	−0.560 (0.210)^**^	−0.219 (0.064)^***^
DOSFREQ	−0.001 (0.000)^***^	−0.0005 (0.000)^***^	−0.001 (0.000)^**^	−0.001 (0.000)^***^	−0.003 (0.001)^***^	−0.001 (0.000)^**^
NONCLINICAL_SELF	0.167 (0.037)^***^	0.108 (0.025)^***^	0.112 (0.038)^**^	0.210 (0.039)^***^	0.424 (0.128)^***^	0.146 (0.045)^**^
NONCLINICAL_SUPV	−0.122 (0.028)^***^	−0.078 (0.019)^***^	−0.067 (0.025)^**^	−0.120 (0.030)^***^	−0.124 (0.093)	−0.098 (0.037)^**^
DDA_MODERATE1	0.391 (0.033)^***^	0.239 (0.033)^***^	0.214 (0.074)^**^	0.437 (0.038)^***^	1.210 (0.150)^***^	0.264 (0.050)^***^
DDA_MODERATE2	−0.267 (0.042)^***^	−0.171 (0.032)^***^	−0.167 (0.059)^**^	−0.300 (0.046)^***^	−1.058 (0.141)^***^	−0.075 (0.061)
DDA_SEVERE	−0.525 (0.039)^***^	−0.336 (0.044)^***^	−0.308 (0.103)^**^	−0.588 (0.044)^***^	−1.376 (0.184)^***^	−0.324 (0.057)^***^
RAE_MODERATE	0.169 (0.034)^***^	0.108 (0.024)^***^	0.111 (0.041)^**^	0.212 (0.038)^***^	0.522 (0.141)^***^	0.067 (0.043)
RAE_SEVERE	−0.743 (0.039)^***^	−0.461 (0.056)^***^	−0.406 (0.138)^**^	−0.869 (0.044)^***^	−2.342 (0.215)^***^	−0.204 (0.059)^***^
COST	−0.008 (0.001)^***^	−0.005 (0.001)^***^	−0.004 (0.001)^**^	−0.0118 (0.040)^***^	−0.012 (0.002)^***^	−0.008 (0.001)^***^
A	−0.054 (0.054)	−0.033 (0.034)	0.002 (0.025)	−0.073 (0.066)	0.370 (0.274)	−0.122 (0.073)!
Entropy$\left(\widehat{\uptheta_1},\widehat{\uptheta_2}\right)$	―	―	(1.026, 0.717)	―	―	
${\widehat{\alpha}}_0$(FEMALE)	―	0.170 (0.035)^***^	―	―	―	
${\widehat{\alpha}}_1$(VOCATIONAL)	―	−0.230 (0.085)^**^	―	―	―	
${\widehat{\alpha}}_2$(GCSEs_O + A)	―	−0.199 (0.054)^***^	―	―	―	
${\widehat{\pi}}_c$	―	―	―	―	0.49	0.51
${\widehat{\gamma}}_c$(VOCATIONAL)	―	―	―	―	−0.892 (0.324)^**^	—
${\widehat{\gamma}}_c$(GCSEs_O + A)	―	―	―	―	0.577 (0.203)^**^	—
${\widehat{\gamma}}_c$(FEMALE)	—	—	—	—	0.285 (0.122)^*^	—
${\widehat{\gamma}}_c$(RESPONDENTAGE)					0.024 (0.009)^**^	
Log-likelihood (AIC)	−2855.357 (5736.713)	−2812.19 (5670.381)	−2837.971 (5670.381)	−2782.51 (5611.019)	−2652.879 (5379.757)	

*Notes*: SE = standard error. For the HMNL and LCMNL models, we report *selected* effects of respondent-characteristics on the scale-parameter and latent-class membership. MMNL model was estimated using 500 Halton draws of correlated normally-distributed coefficients for the variables A, INTRAVENOUS and SUBCUTANEOUS and a log-normal distributed cost coefficient. *** *p* < 0.001 ** *p* < 0.01 * *p* < 0.05! *p* < 0.10. AIC = Akaike Information Criterion

That said, the other models provide useful insights on the choice behaviour of respondents. For example, a Lagrangian Multiplier test for heteroskedastic errors in the HMNL model showed statistically significant unobserved variation that is explained by gender, age, education and employment status. Other respondents’ characteristics: prior illness within the past year and household-income were only statistically significant contributors to unobserved heterogeneity at the 10% level. (Note that Table 4 only reports selected findings on the set of contributors to unobserved heterogeneity in the HMNL model.) Similarly, a Lagrangian Multiplier test for heteroskedastic errors in the EMNL model showed statistically significant unobserved variation.^3^ However, the statistically insignificant entropy parameters ($\widehat{\uptheta_1},\widehat{\uptheta_2}$) of the EMNL model, with $\widehat{\uptheta_1}>0$, suggested that respondent fatigue (perhaps offset by learning effects) could be ignored as plausible explanations for the sequence of choices observed. We believe this justifies our decision to block the experimental designs underlying the survey questionnaires. That aside, the improvement in log-likelihoods observed with the MMNL model, over and above that of the MNL model, confirm there are some significant variations in and correlations between the coefficients drawn from the continuous mixture distribution. This heterogeneity and correlations in preferences, however, can be captured equivalently by the LCMNL model.

Focusing on the results of the LCMNL model, we observed the probability that any individual belongs to the first subgroup (latent-class 1) is determined by age, gender, and education; and not household income or prior illness suffered in the previous year. Conditional on membership of latent-class 1, the average or representative survey respondent is indifferent to needle-free modes of drug administration when compared with intravenous or subcutaneous routes conditional on the other attributes. Respondents are indifferent in the sense that coefficients for the INTRAVENOUS and SUBCUTANEOUS variables were not statistically different from zero. We interpret this to mean respondents are informed enough to know that, in some disease states, needle-free routes may not be the best or a feasible method of drug administration. On the other hand, respondents, on average, show a negative preference for intramuscular modes of drug delivery when compared with needle-free routes, perhaps because of the pain involved. Similarly, we observed a negative preference for drug administration modes that involve higher dosing frequency albeit the magnitude of the effect was small and close to zero. We observed also a positive preference for self-administration within a non-clinical setting and a negative statistically insignificant preference for drug administration in non-clinical settings under supervision. A probable explanation for this result is that if administering a drug requires supervision by a qualified healthcare professional, then one might be better off having the drug administered in a clinical setting.

Our results show a positive preference for “moderate but manageable” disruptions to daily activities and a negative preference for “severe” or “moderate but unmanageable” disruptions to daily activities. This suggests that respondents did take into account the hypothetical nature of the choice tasks: although possible (in the future), a mode of drug administration that is associated with zero disruption to daily activities may not be currently available or technologically feasible. Similarly, we observed a positive preference for modes of drug administration associated with a “moderate” risk of adverse events and a negative preference for modes of drug administration associated with “severe” risk of adverse events. Again, we observed, on average, some kind of mental accounting of the fact that a mode of drug delivery that has a zero risk of adverse events may not be available or technologically feasible.

Conditional on membership of latent-class 2, we observe similar choice patterns with the following exceptions. First, coefficients for the variables for moderate risk of disruptions-to-daily-activities and moderate risk of adverse events were not statistically significant. That respondents belonging to latent-class 2 show indifference to these attribute-levels provides further support to our argument that respondents may have, in their decision choices, considered that drug delivery modes with zero disruptions to their daily activities and/or zero risk of adverse events are perhaps unavailable even though they are desirable. Second, the magnitudes of the coefficients for the INTRAVENOUS and SUBCUTANEOUS variables indicate a positive preference for these modes of drug administration relative to needle-free routes of administration. But since these effects are not statistically different from zero, we maintain the argument that respondents are generally indifferent to the choice between needle-free and intravenous/subcutaneous routes of drug administration.

For both latent-classes, we observe a small but statistically significant coefficient for the cost attribute. This suggests that our survey respondents have price inelastic “demands” for the attributes of drug administration we investigated in response to any (out-of-pocket) costs of accessing healthcare. This probably reflects two things. One, the fact that the UK NHS provides tax-funded insurance protection against the financial risks of ill health; and two, that the costs in question are by and large ‘unavoidable’: without spending resources on some form of a vehicle for administering a drug, patients will be unable to realize the direct health benefits offered by that drug.

### Patient benefits and cost savings

Table 5 shows MWTP estimates for each of the non-cost attributes studied and the associated confidence intervals around these estimates. A positive $\widehat{\mathrm{MWTP}}$ indicates a preference for an attribute taking into account the associated cost, whilst a negative $\widehat{\mathrm{MWTP}}$ indicates a dispreference. As observed in Table 4, there are subtle differences in the MWTP estimates obtained from the different econometric models. We focus on estimates from the LCMNL model as this provided the best fit with the choice data collected. This shows a statistically significant and substantial willingness-to-pay for drug delivery modes that are associated with “moderate” disruption to daily activities and “moderate” risk of adverse events. There is also a statistically significant and substantial willingness-to-pay to avoid drug delivery modes that are associated with “moderate but unmanageable” or “severe” disruptions to daily activities. Similarly, there is a statistically significant and substantial willingness-to-pay to avoid drug delivery modes that are associated with “severe” risk of adverse events. Further, there is a statistically significant and substantial willingness-to-pay to avoid drug administration via the intramuscular route.

**Table 5:** Marginal willingness-to-pay estimates

	MNL model	HMNL model	EMNL model	MMNL model	LCMNL model
Variables:	$\widehat{\mathrm{MWTP}}\left(95\%CI\right)$	$\widehat{\mathrm{MWTP}}$(95% CI)	$\widehat{\mathrm{MWTP}}\left(95\%CI\right)$	$\widehat{\mathrm{MWTP}}\left(95\%CI\right)$	$\widehat{\mathrm{MWTP}}\left(95\%CI\right)$
INTRAVENOUS	−2.12 (−4.09, −0.16)^*^	1.17 (−0.74, 3.08)	3.43 (1.73, 5.14)^*^	2.31 (−2.86, 7.49)^*^	5.85 (−50.38, 62.07)
SUBCUTANEOUS	2.35 (1.37, 3.32)^*^	2.60 (1.58, 3.63)^*^	0.23 (−0.52, 1.03)	−8.33 (−10.31, −6.34)^*^	2.42 (−7.76, 12.59)
INTRAMUSCULAR	−17.43 (−18.65, −16.22)^*^	−19.19 (−20.41, −17.97)^*^	−26.08 (−27.15, −25.01)^*^	−9.39 (−11, −7.77)^*^	−40.63 (−72.47, −8.80)^*^
DOSFREQ	−0.08 (−0.09, −0.08)^*^	−0.09 (−0.094, −0.085)^*^	−0.12 (−0.13, −0.12)^*^	−0.10 (−0.10, −0.09)^*^	−0.23 (−0.30, −0.17)^*^
NONCLINICAL_SELF	21.45 (20.51, 22.39)^*^	22.41 (21.48, 23.35)^*^	26.35 (25.55, 27.16)^*^	31.01 (29.87, 32.14)^*^	28.57 (21.40, 35.74)^*^
NONCLINICAL_SUPV	−15.45 (−16.06, −14.83)^*^	−15.74 (−16.36, −15.11)^*^	−15.46 (−15.95, −14.96)^*^	−19.83 (−20.64, −19.02)^*^	−15.53 (−26.04, −5.03)^*^
DDA_MODERATE1	50.09 (48.91, 51.27)^*^	49.40 (48.15, 50.65)^*^	50.14 (49.02, 51.27)^*^	58.88 (57.65, 60.11)^*^	84.71 (67.59, 101.82)^*^
DDA_MODERATE2	−34 (−35.15, −32.85)^*^	−35.24 (−36.51, −33.97)^*^	−38.86 (−39.89, −37.83)^*^	−43.13 (−44.35, −41.92)^*^	−58.84 (−72.28, −45.40)^*^
DDA_SEVERE	−66.36 (−67.74, −64.99)^*^	−68.64 (−70.14, −67.14)^*^	−71.55 (−72.80, −70.30)^*^	−73.64 (−75.15, −72.14)^*^	−94.50 (−117.91, −71.10)^*^
RAE_MODERATE	22.06 (21.20, 22.92)^*^	22.64 (21.74, 23.53)^*^	26.37 (25.59, 27.14)^*^	30.32 (29.23, 31.41)^*^	23.49 (17.29, 29.70)^*^
RAE_SEVERE	−94.65 (−96.64, −92.65)^*^	−94.77 (−96.85, −92.70)^*^	−94.75 (−96.58, −92.93)^*^	−116.08 (−117.65, −114.51)^*^	−130.32 (−165.12, −95.53)^*^

*Note*s: The 95% CIs above are “standard or classical confidence intervals” calculated using 100 bootstrapped replicates of MWTP. This is because accurate, less-erratic and reliable “bootstrap confidence intervals” require replications in the order of 1000, which would have been computationally demanding and time consuming [32]. The confidence intervals reported are therefore not exact. * indicates confidence interval does not include zero

To evaluate the welfare change, i.e., the intangible benefits from manufacturing drugs in a patient-friendly manner, we considered the following. A given biologic drug *C* can be manufactured in two ways (C1 and C2). Assume, as we did in the survey, that both versions of the drug have the same molecule, efficacy and safety profile. In option C1, the drug can be manufactured for intravenous administration in clinical settings, and this mode of drug delivery is associated with “severe” risk of adverse events and “severe” disruptions to patients’ daily activities. Option C2 is where a drug is manufactured for subcutaneous self-administration in non-clinical settings and this mode of drug delivery is associated with “moderate” risk of adverse events and “moderate” disruptions to patients’ daily activities. In this case, we can compute the expected compensating variation ($\widehat{ECV}$) using eq. (8). Since household-income categories had no statistically significant effect on class membership in our preferred LCMNL model, we do not differentiate our $\widehat{ECV}$ by household-income category.

Based on the MNL model, switching from option C1 to C2 yields a welfare gain ($\widehat{ECV}$ per patient per unit administration) of -£296 (95% CI: −£302 to -£289). Based on the MMNL model, $\widehat{ECV}$ is: −£364 (95% CI: −£370 to -£358). Based on the LCMNL model with two latent-classes, and unconditional on latent-class membership, we obtained $\widehat{ECV}$ of -£435 (95% CI: −£524 to -£346). Note that the ECV is a measure of welfare gain, not welfare loss. The negative sign reflects the fact that ECV is the amount of money that has to be *taken from* the state of having option C2 so that the average respondent will be indifferent to option C1 (see equation 8). Note also that with our choice data failure to control for preference heterogeneity in the MNL model leads to an underestimation of welfare change. The same argument may not be applicable to other choice data. But how does our ECV estimates (of intangible benefits to patients) compare with savings in drug administration costs to a healthcare payer?

To answer this question, we used a regression-based algorithm to predict the cost of UK NHS resources that will be consumed in administering drugs C1 and C2. Details of this algorithm will be found elsewhere [4]. We maintained all previous assumptions made in computing estimates of ECV, and added the following. One, both drugs C1 and C2 are indicated for management of a chronic illness; two, product C2 is sold bundled with some of the equipment and consumables used in drug administration; and three, a single full treatment course of C1 over a year requires 10 unit administrations whilst a single full treatment course of C2 requires 5 unit administrations over a year. Direct monetary costs of administering drugs C1 and C2 were then estimated as:

$$ \ln AD\widehat{\mathrm{MINC}}{OST}_{C1}=7.1499-3.2026(0)-5.2737(0)+.428(10)+.404(0)-.2896(1)-.00106\left({10}^2\right)-.3173(10)(1) $$

$$ AD\widehat{\mathrm{MINC}}{OST}_{C1}= \exp (7.8613)\bullet \widehat{\Phi}\left(=1.0792\right)=\mathrm{\pounds}2800.41 $$

$$ \ln AD\widehat{\mathrm{MINC}}{OST}_{C2}=7.1499-3.2026(1)-5.2737(0)+.428(5)+.404(1)-.2896(1)-.00106\left({5}^2\right)-.3173(5)(1) $$

$$ AD\widehat{\mathrm{MINC}}{OST}_{C2}= \exp (4.6099)\bullet \widehat{\Phi}\left(=1.3799\right)=\mathrm{\pounds}138.64 $$

where $\hat{\Phi} =$ 1.0792 and 1.3799 are the subgroup-specific smearing factors for intravenous and subcutaneous products respectively.

The estimates above yield a cost saving of roughly £2662 per patient per year; or £532 per patient per unit administration of switching from C1 to C2. This is comparable to the absolute value of $\widehat{ECV}$: £435 derived from the LCMNL model.

## Discussion

The non-zero MWTP and ECV estimates reported above provide a monetary measure of the “clinical usability” of a drug – where clinical usability has to do with the mode of drug administration as separate from considerations of efficacy, safety and/or value-for-money. Some might consider MWTP and ECV as old-fashioned, redundant metrics of welfare change – arguing that it is better to evaluate predicted choice probabilities for a selected group of products (bundles of attributes). However, such discrete demand analyses will not allow us to compare the monetary value of the intangible benefits from making patient-friendly medicines with the monetary savings in drug administration costs.

From our analyses, we found that the monetary value of the intangible benefits (from satisfied patients’ preferences) is in the same order of magnitude as savings on the direct monetary costs of resources healthcare providers spend on drug administration. Our results also indicate a strong positive preference for modes of drug administration that are associated with some but not significant risks of adverse events and/or disruptions to patients daily activities. They also show a positive preference for self-administration of drugs in non-clinical settings – and a negative preference for drug administration in clinical settings or non-clinical settings under the supervision of a qualified healthcare professional. Advances in biopharmaceutical manufacturing such as pre-filled syringes, auto-injectors and pen injectors and other innovations that reduce the risk of adverse events and/or disruptions to daily activities clearly hit with these observations.

This argument, however, assumes that the state of biopharmaceutical manufacturing and formulation science is mature enough to support the desired innovations in making patient-friendly medicines and/or that the profit signals are strong enough to get manufacturers to consider end-user preferences. There might be, of course, practical manufacturing challenges that militate against reverse engineering product option C1 to C2. Nevertheless, our estimates indicate there are substantial benefits from developing patient-friendly drug delivery systems if the underlying formulation and manufacturing science makes it possible to do so. If these societal benefits are considered important by policy makers, then there is a case for public interventions to encourage manufacturing research in an attempt to achieve the desired goal of producing clinically usable medicines and drug delivery devices. With the right pricing and reimbursement environment, an additional incentive for manufacturers to consider the preferences of end-users may come from attempts to differentiate products in order to maintain or increase market shares.

For any given cohort of patients (end-users), a drug product that closely matches the preferences of the average representative end-user or consumer should enjoy higher demand volumes (keeping prices unchanged). Product differentiation along the lines of satisfying end-user preferences for the mode of drug administration may indeed create brand loyalty without manufacturers engaging in academic detailing or direct-to-consumer advertising. We would also expect additional demand inducement where manufacturing a drug product in a patient-friendly manner, amplifies the (incremental) direct health benefits derived from that drug. This is particularly important considering healthcare payers and providers’ requirements for estimates of cost-effectiveness from manufacturers to demonstrate product value. Our ECV estimates measure indirect or intangible benefits assuming direct health benefits remain the same. So if healthcare payers and providers are willing to pay for the value of the drug delivery mode, and the discounted present value of private producer surplus of developing patient-friendly drug delivery systems (relative to other investment opportunities) is positive, then manufacturers should consider the switch from C1 to C2.

As with all research, a number of limitations apply to the arguments above.

First, the list of attributes evaluated in this study was taken from a selective literature review. Ideally, one would want to supplement this literature review with interviews and/or focus group discussions involving end-users. Given the time and resources available for this study, we were not able to apply these qualitative methods – which are of most value where there is a lack or dearth of existing (grey) literature. We therefore make no claim here that the selected set of attributes and attribute-levels are exhaustive of all characteristics of all possible modes of drug administration. We believe, however, that the selected attributes and attribute-levels in Table 1 are realistic, relevant and suited for investigating the gross welfare benefits (consumer surplus) from manufacturing patient-friendly medicines. Second, the levels “none”, “moderate” and “severe” for the attribute risk-of-adverse-events, for example, will be understood differently by different respondents with different backgrounds. The attribute-levels were chosen to represent a natural categorical ordering of risk or severity; but the strength of respondents’ preferences could be influenced by differences in attribute perception. Unfortunately, we did not include variables constructed to measure attribute perceptions in our analysis. Differences in attribute perception will appear as preference heterogeneity in the MMNL and LCMNL models we estimated. Or, with preferences restricted to be the same, appear as unobserved heterogeneity in the HMNL and EMNL models. We cannot therefore make any statements about the precise impacts of differences in attribute perception on choices. What we know is: age, gender and education affect variation in individual preferences; and the same set of respondent characteristics plus employment status influence unobserved heterogeneity.

Third, it might be argued that our ECV estimates depend on the cost levels chosen. However, Hanley et al. [29] have shown that using different levels for the cost attribute may not result in statistically significant differences in estimates of welfare change albeit there is a possibility that such differences might be significant when the ECV estimates are employed in cost-benefit analyses, for example. See also Slothuus et al. [30]. In our study, we believe that the chosen cost range with an upper limit of £100 adequately captures out-of-pocket access costs that NHS patients are most likely to pay. Considering also the near zero coefficients, we will argue that cost levels beyond £100, and any non-linearity in the cost-attribute effects, are unlikely to change the arguments above. Fourth, some might argue that $\widehat{ECV}$ derived from the LCMNL model suffers from ecological fallacy – as they are based on the average weighted coefficients over two latent-classes. For that matter (erroneous) conclusions that apply at the aggregate level may not apply at the latent-class level. However, we do not know a priori which latent-class a given respondent belongs to. Since we cannot assume fixed class membership, the reported $\widehat{ECV}$, which is unconditional on class membership, is a valid measure of welfare change.

Finally, we have only evaluated the preferences of mostly healthy people from the UK general public at a given point in time. If preferences change over time, our estimates may no longer be valid albeit we will not expect any dramatic differences from what we have reported. A possible avenue for future research is to repeat the analysis here using a panel data of discrete choices collected over time. That aside, it is well known that end-user preferences for healthcare interventions in healthy states are not the same as when they are in sick states – and that people make decisions behind a “veil of experience”, i.e., they prefer products and services that they have previously experienced [31]. Hence, our sample which was dominated by healthy people may bias our estimates. We did indeed recognize the issue and it is for this reason we linked the variable PRIOR_ILLNESS to the scale parameter in the HMNL model – to partially account for health-state-dependent preferences and the experience-good features of healthcare. What is more the variable for prior illness was not a statistically significant predictor of preference heterogeneity in the LCMNL model. We did not ask survey respondents the type of illness (acute or chronic) they had experienced. The variable for prior illness therefore is crude and it says nothing about the number of visits to a health facility in the past year or the severity of illness and whether this affects respondents’ cognitive abilities. It is then possible that specific patient-populations (for example, those suffering from Alzheimer’s, diabetes or some form of cancer) may have preferences that differ from that of the sample we studied. We leave this issue of health-state-dependent preferences for future research.

## Conclusions

In this study, we attempted to estimate the monetary value of end-user preferences for a generic set of attributes of different modes of drug administration. We found a non-trivial marginal willingness-to-pay for drug delivery systems associated with zero or moderate risk of adverse events and/or disruption to patients’ daily activities. We also found a high marginal willingness-to-pay for self-administration of drugs in non-clinical settings. In addition, we estimated that the monetary value of making patient-friendly medicines could be as large as the savings on direct monetary costs of drug administration to healthcare payers and providers. We argue that as long as there is recognition of the value of the drug delivery mode (besides the value of drug molecules in improving health outcomes); and the underlying manufacturing science is capable, a patient-centred approach to producing beneficial drugs and drug delivery systems should be encouraged and pursued.

## Footnotes

1 Note that our simulated discrete-choice data were not derived from known utility functions or a known ***β*** vector. Our interests are in the robustness of the attribute-coefficients ($\widehat{\boldsymbol{\beta}}$) and not how $\widehat{\boldsymbol{\beta}}$ closely approximates prior or “true” values of ***β***. The lower standard errors and high number of statistically significant coefficients, obtained from the same MNL model estimated with the same simulated dataset, confirms the efficiency gain from our chosen experimental design.

2 In determining *C**, we estimated a number of LCMNL models with 2–10 latent-classes. To increase the speed of computation, we estimated these models without the **Z** variables in the class-membership function, i.e., equation (6). We found the closest competitor to the LCMNL with two-classes (cAIC = 5522.461, BIC = 5495.461) was an LCMNL with four classes (cAIC = 5528.196, BIC = 5473.196). We chose the two-class LCMNL model on the basis of precision loss in the estimated coefficients: the fourth latent-class of the LCMNL model with four-classes had no statistically significant coefficients.

3 Because the measure of entropy is derived from choice probabilities predicted from the MNL model, it is in effect an “endogenous” explanatory variable that might bias the coefficients of the EMNL model. In Table 4, we observe that, even if these coefficients are biased, they are consistent with that of the MNL and HMNL models. What is more, the primary purpose of the EMNL model is to assess respondents’ (fatigued) reactions to the choice tasks, i.e., we are interested mostly in equation (4) and not equation (3).
